# Changes in uPA, PAI-1, and TGF-β Production during Breast Cancer Cell Interaction with Human Mesenchymal Stroma/Stem-Like Cells (MSC)

**DOI:** 10.3390/ijms20112630

**Published:** 2019-05-28

**Authors:** Catharina Melzer, Juliane von der Ohe, Hannah Otterbein, Hendrik Ungefroren, Ralf Hass

**Affiliations:** 1 Biochemistry and Tumor Biology Lab, Department of Obstetrics and Gynecology, Hannover Medical School, 30625 Hannover, Germany; melzer.catharina@mh-hannover.de (C.M.); Ohe.Juliane.von.der@mh-hannover.de (J.v.d.O.); 2 First Department of Medicine, UKSH, Campus Lübeck, 23538 Lübeck, Germany; hannahotterbein@web.de (H.O.); Hendrik.Ungefroren@uksh.de (H.U.); 3 Department of General Surgery, Visceral, Thoracic, Transplantation and Pediatric Surgery, UKSH, Campus Kiel, 24105 Kiel, Germany

**Keywords:** mesenchymal stem cells, breast cancer, cancer cell interaction, co-culture, tumor microenvironment, uPA, PAI-1, TGF-β

## Abstract

The interactions of cancer cells with neighboring non-malignant cells in the microenvironment play an important role for progressive neoplastic development and metastasis. Long-term direct co-culture of human MDA-MB-231^cherry^ breast cancer cells with benign human mesenchymal stroma/stem-like cells (MSC) MSC544^GFP^ stably expressing mCherry and eGFP fluorescence proteins, respectively, was associated with the formation of three-dimensional (3D) tumor spheroids in vitro. The quantification of the breast tumor marker urokinase plasminogen activator (uPA) in mono-cultured MDA-MB-231 cells revealed an approximately 14-fold enhanced expression when compared to five different normal human MSC mono-cultures. Moreover, uPA levels in 3D tumor spheroids remained elevated 9.4-fold above the average of five different human MSC cultures. In contrast, the expression of the corresponding plasminogen activator inhibitor type-1 (PAI-1) declined by 2.6-fold in the breast cancer cells and was even further reduced by 3.2-fold in the MDA-MB-231^cherry^/MSC544^GFP^ 3D co-culture spheroids when compared to the various MSC populations. The supportive data were obtained for the production of TGF-β1, which is an important growth factor in the regulation of tumor growth and metastasis formation. Whereas, TGF-β1 release in MDA-MB-231^cherry^/MSC544^GFP^ co-cultures was elevated by 1.56-fold as compared to MSC544 mono-cultures after 24 h; this ratio further increased to 2.19-fold after 72 h. Quantitative PCR analyses in MSC544 and MDA-MB-231 cells revealed that MSC, rather than the breast cancer cells, are responsible for TGF-β1 synthesis and that TGF-β1 contributes to its own synthesis in these cells. These findings suggested potential synergistic effects in the expression/secretion of uPA, PAI-1, and TGF-β during the co-culture of breast cancer cells with MSC.

## 1. Introduction

In the course of tumor development and formation of metastases, cancer cells change cell fate, adhesive properties, cell movements, and motility during interactions with the microenvironment [1]. The cancer cells interact with components of the extracellular matrix and adjacent stromal cells, such as tumor-associated macrophages, cancer-associated fibroblasts, different immune cell subsets (lymphocytes, natural killer cells, dendritic cells), adipocytes, endothelial cells, pericytes, and mesenchymal stroma/stem-like cells (MSC) [2,3,4]. Moreover, reciprocal release and uptake of extracellular vesicles and factors, including proteinases and growth factors, contribute to cancer cell interactions. Among those are the serine proteinase urokinase plasminogen activator (uPA), its inhibitor plasminogen activator inhibitor type-1 (PAI-1), and the regulatory cytokine transforming growth factor-β (TGF-β).

Physiological uPA can exist in different forms, including a high molecular weight (HMW)-uPA with two amino acid chains that were connected by a disulfide bridge and a low molecular weight (LMW)-uPA form exhibiting proteolytic activity. The substrates for uPA are several components of the extracellular matrix and a predominant target of uPA is represented by plasminogen with the subsequent conversion to plasmin upon uPA-mediated cleavage of a specific Arg-Val amino acid bond. The activation of uPA and subsequent degradation of target proteins can further relay proteolytic cascades, which also play an important role during neoplastic development [5].

PAI-1, which is also known as serpin E1, functions as a serine proteinase inhibitor (serpin) and it predominantly targets uPA besides tissue plasminogen activator and some distinct matrix metalloproteinases [6]. The inhibitory activity is mediated by either direct binding of PAI-1 to the active site of uPA and/or by the association of PAI-1 with the uPA/uPAR receptor complex. Both uPA and PAI-1 play an important role as prognostic factors in breast cancer [7]. In clinical practice, the determination of uPA and PAI-1 (by ELISA) is used for nodal negative mammary carcinoma with intermediate grading, whereby uPA and PAI-1 levels are separately evaluated to estimate the necessity of chemotherapy among other parameters at the highest level of evidence.

The three isoforms of TGF-β (TGF-β1-3) belong to the TGF-β/BMP/activin superfamily of the growth and differentiation factors. TGF-β1 controls proliferation, differentiation, apoptosis, and cell motility, and it is produced by both MSC and breast cancer cells. It acts as a tumor suppressor in normal epithelial cells and premalignant tumor stages, but it can simultaneously promote tumor progression through tumor-cell-autonomous and tumor-stroma interactions which can evolve during immune evasion, stimulation of angiogenesis, and metastatic development [8]. The TGF-β propeptide is part of a complex together with latent TGF-β binding protein (LTBP), termed latency-associated peptide (LAP), prior to its secretion. Still inside the cell, the propeptide is cleaved from the precursor, but it remains associated with LAP via strong non-covalent interactions. The uPA-generated plasmin serum proteinase as well as the matrix metalloproteinases MMP-2 and MMP-9 can catalyze the release of active TGF-β from LAP by proteolytic degradation [9].

In the present work, we demonstrate the alterations in the production of uPA, PAI-1, and TGF-β during co-culture of MSC with highly malignant breast cancer cells that may support continued tumor growth.

## 2. Results and Discussion

**Figure 1:**
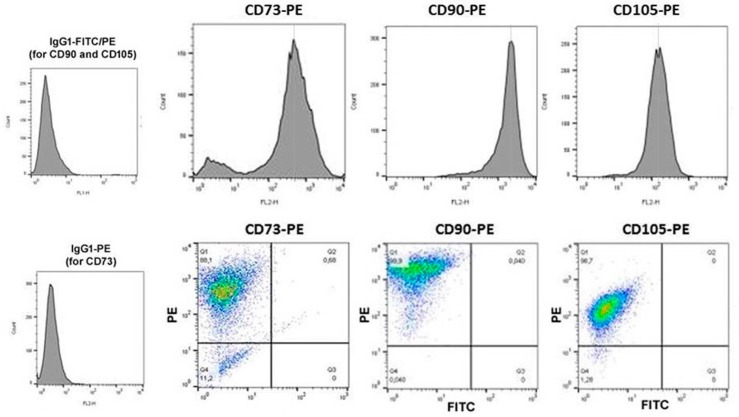
Expression of typical mesenchymal stroma/stem-like cells (MSC) markers in MSC544. Isolated human MSC544 from a patient with a benign phyllodes tumor were long-term cultured (until P15 (for CD73) and P22 (for CD90 and CD105)), whereby the presence of surface markers that are associated with a mesenchymal stroma-/stem-like cell phenotype could be characterized.

We now tested the role of a MSC population derived from a benign human phyllodes tumor, while previous works demonstrated various types of indirect and direct interactions between normal primary human MSC and breast cancer cells [4,10]. In contrast to our normal primary human MSC that maintain proliferative capacity, cell fate, and marker expression for up to 10 cell passages [11,12,13], the neoplastic tissue-derived MSC544 continued to grow and it maintained the expression of typical MSC markers beyond passage 10 (P10). Thus, flow cytometry analysis of ecto-5’-nucleotidase (CD73), Thy-1 membrane glycoprotein (CD90), and endoglin (CD105), which is a component of the TGF-β receptor complex, revealed the simultaneous presence on more than 99% of MSC544 after P22, suggesting the long-term maintenance of MSC characteristics (Figure 1).

**Figure 2:**
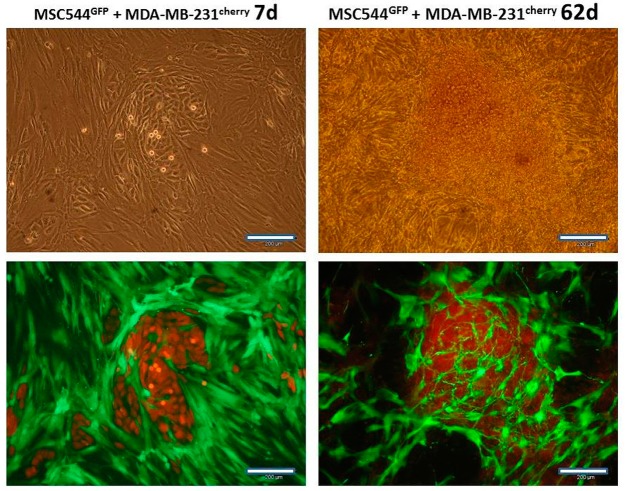
Long-term co-culture of human MSC544^GFP^ with MDA-MB-231^cherry^ breast cancer cells. Phase contrast (upper panel) microscopy, double fluorescence (red + green) microscopy (lower panel, left micrograph), and only green fluorescence microscopy (lower panel, right micrograph) was performed in co-cultures of MSC544^GFP^ with MDA-MB-231^cherry^ breast cancer cells for 7d and 62d, respectively. The highly proliferating and overgrowing breast cancer cells were forming three-dimensional (3D) spheroids together with MSC after 62d whereby the bright cherry fluorescence was partially detectable in the green fluorescence channel (lower panel, right micrograph). Scale bars: 200 µm.

Co-cultures of lentivirus-labeled MSC544^GFP^ populations, together with MDA-MB-231^cherry^, demonstrated breast cancer cell colony patches with surrounding MSC after 7d, as documented by phase contrast and double fluorescence microscopy (Figure 2, left panels). Similar findings were previously observed during the interaction of normal primary human MSC with breast cancer cells [10]. However, recent data have demonstrated that slower proliferating normal primary human MSC are rapidly overgrown by breast cancer cells with less than 1% of total cell counts remaining after 14 days of co-culture both, in vitro and in vivo [14]. In contrast, MSC544^GFP^ also remained proliferation-active in long-term co-culture with breast cancer cells. While co-cultured MDA-MB-231^cherry^ cells had formed three-dimensional (3D) spheroids after 62 days, MSC544^GFP^ established a cellular network surrounding these 3D cell clusters (Figure 2, right panels). Nevertheless, the phase contrast micrograph of the 62 days co-culture revealed the complete coverage with cells (Figure 2, upper panel, right micrograph). While the red fluorescence and the double fluorescence channels revealed red images with indistinguishable morphologies, the corresponding selective cell population micrographs were obtained from the green fluorescent channel (Figure 2, lower panel, right micrograph), which suggested the presence of a majority of MDA-MB-231^cherry^ breast cancer cells with partial detection of a bright cherry cross-fluorescence obtained from the 3D breast cancer spheroids.

**Figure 3:**
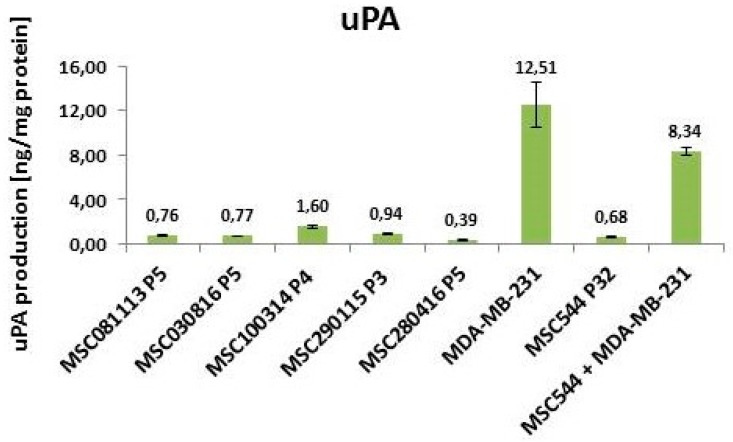
Quantification of urokinase plasminogen activator (uPA) in different MSC cultures and MDA-MB-231 breast cancer cells. Intracellular amounts of uPA were quantified in five different primary MSC cultures at different passages and compared with MSC544 P32. Moreover, the levels of uPA in MSC544 were compared to MDA-MB-231 breast cancer cells and to a 3D spheroid formed after long-term co-culture (62 days) between MSC544^GFP^ and MDA-MB-231^cherry^. Data represent the mean ± s.d. (*n* = 4).

Moreover, the appearance of multiple 3D breast cancer spheroids and disseminated MDA-MB-231 cells in the co-culture suggested the enhanced liberation and distribution of the breast cancer cells. Indeed, previous work has demonstrated changes in these organoid structures by MSC-mediated decrease of E-cadherin in breast cancer spheroids [15,16]. This is also substantiated by high uPA levels in MDA-MB-231 cells (Figure 3), whereby uPA represents one of the serine proteinases that contribute to matrix digestion and thus cellular motility. Previous work suggested that elevated uPA levels in association with the plasminogen activation system correlates with tumor malignancy. Consequently, partial tumor tissue degradation by the urokinase system, followed by the liberation of cancer cells, facilitates tissue invasion and contributes to metastasis, which therefore may represent a potential drug target using anticancer agents [17]. Indeed, the reduction of Migfilin, β-catenin, and uPA via the depletion of the regulatory vasodilator-stimulated phosphoprotein inhibited tumor spheroid invasion of MDA-MB-231 cells [18]. More specifically, the down-modulation of uPA by targeting the uPA 3’ untranslated region with microRNA-645 reduced the invasive growth of MDA-MB-231 cells [19].

In contrast to the high uPA levels of 12.51 ng/mg protein in MDA-MB-231 cells, five different primary MSC populations exhibited uPA levels, with the highest amount reaching 1.6 ng/mg protein in MSA100314 P4 (Figure 3). Thus, the neoplastic tissue-derived MSC544 P32 displayed 0.68 ng uPA/mg protein, which is in line with the uPA values that were obtained for the other primary MSC populations. Together with the non-tumorigenic state of normal MSC, these findings suggested that the constitutively low uPA levels in primary MSC and MSC544 do not significantly contribute to the invasive properties of these cell populations.

Of interest, the co-cultures of MSC544^GFP^, together with MDA-MB-231^cherry^, maintained high uPA levels of 8.34 ng/mg protein in the 3D spheroids, indicating the presence of invasive potential in these organoids (Figure 3). This is substantiated by previous findings that co-cultures of human MSC with breast cancer cells, including MDA-MB-231, closely interact with each other and they are associated with increased proliferative capacity in vitro when compared to the corresponding mono-cultures [20,21]. Moreover, these co-cultures also contribute to enhanced in vivo tumor growth that I associated with elevated formation of metastases and a potential generation of breast cancer stem cells, which may also involve TGF-β, Rac1, and Rac1b signaling [22,23,24,25]. Further studies revealed that cytokines, including MSC-released CC-motif chemokine ligand 5 (CCL5 = RANTES), promote tumor growth and metastases formation upon cross-talk with breast cancer cells [26]. With respect to RANTES, quantitative real-time RT-PCR (qPCR) analyses revealed the downregulation of the mRNA in MDA-MB-231 cultures after eight days versus day 1 (71%), and a strong upregulation in MSC544 cultures (97.8-fold), whereas only small changes were detected in the co-cultures (Figure S1, upper panel). Interestingly, the reverse was true for epidermal growth factor (EGF) mRNA, upregulation in MDA-MB-231 (19-fold) and downregulation in MSC544 cultures to undetectable levels after eight days of culture. In the co-cultures, the EGF transcripts were two-fold higher on day 8 as compared to day 1 (Figure S1, lower panel).

**Figure 4:**
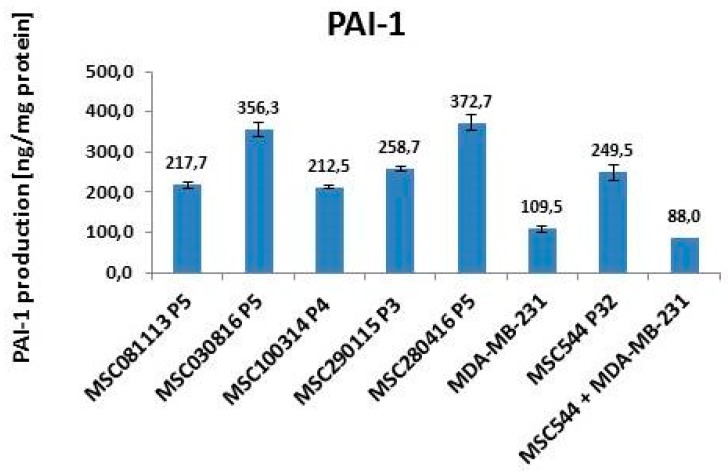
Quantification of PAI-1 in different MSC cultures and MDA-MB-231 breast cancer cells. The intracellular levels of PAI-1 were quantified in five different primary MSC cultures at different passages and compared with MSC544 P32. Moreover, the amount of PAI-1 in MSC544 was compared to MDA-MB-231 breast cancer cells and to a 3D spheroid that formed after long-term co-culture (62 d) between MSC544^GFP^ and MDA-MB-231^cherry^. Data represent the mean ± s.d. (*n* = 4).

A more reciprocal expression pattern when compared to uPA amounts in MSC and MDA-MB-231 cells is displayed by the corresponding inhibitor PAI-1. The five primary human MSC cultures revealed high constitutive PAI-1 values between 212.5 ng/mg protein for MSC100314 P4 and 372.7 ng/mg protein for MSC280416 P5. Within the range of these MSC values, the long-term cultured neoplastic tissue-derived MSC544 at P32 displayed 249.5 ng/mg PAI-1 protein (Figure 4).

In contrast, PAI-1 expression in MDA-MB-231 cells was much lower, reaching only 109.5 ng/mg protein (Figure 4). Previous work in the non-metastatic MCF-7 breast cancer cell line demonstrated low PAI-1 and uPA levels at the detection limit [27]. According to the function of PAI-1 as an inhibitor for uPA, low PAI-1 amounts in the breast cancer cells would be expected to only partially block high uPA activities. As a consequence, uPA remains more active in MDA-MB-231 cells, e.g., for the degradation of extracellular matrix and liberation of cancer cells. These effects are even enhanced in co-cultures of the breast cancer cells, with MSC544 reaching PAI-1 values of 88 ng/mg protein (Figure 4). Together, these findings suggest synergistic interactions during co-cultures of the breast cancer cells with MSC544 and the formation of the 3D tumor spheroids, whereby PAI-1 expression was much lower when compared to that detected in the corresponding MDA-MB-231 and MSC544 mono-cultures. Therefore, mutual interactions between the cancer cells and MSC eventually impair tissue homeostasis and potentially promote neoplastic development and progression [28].

**Figure 5:**
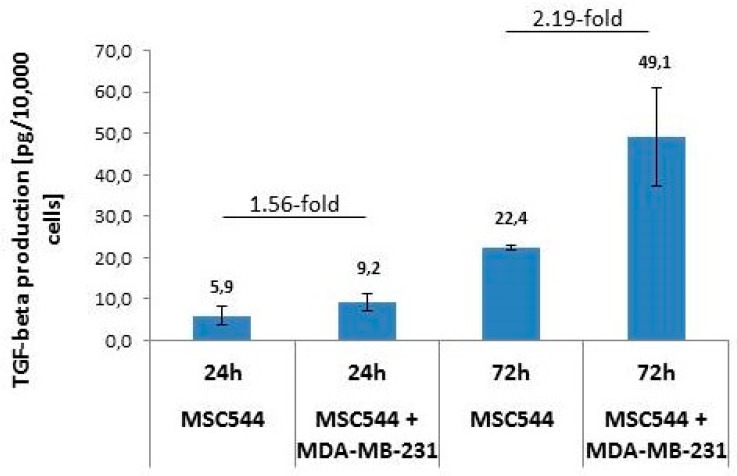
Quantification of secreted bioactive cytokine transforming growth factor-β1 (TGF-β1) in MSC544 mono-cultures and co-cultures of MSC544 with MDA-MB-231 cells. Release of active TGF-β1 from human MSC544 cells (10,000 cells/24-well) was quantified by TGF-β1-specific ELISA and compared to TGF-β1 production of co-cultures between MSC544 and MDA-MB-231 cells (5000 + 5000 cells/24-well) after 24 and 72 h, respectively. Data represent the mean ± s.d. of three independent experiments.

**Figure 6:**
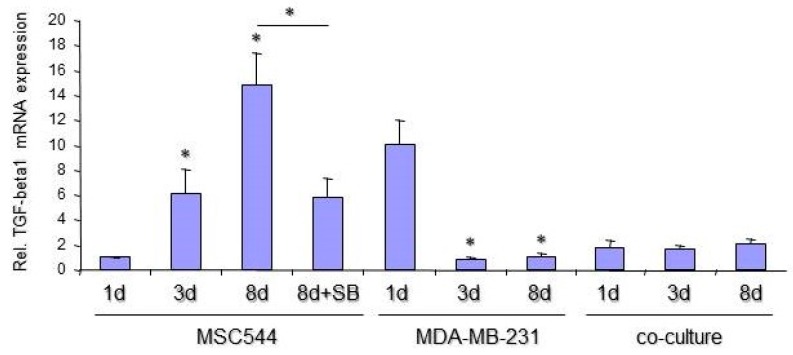
Quantification of TGF-β1-specific mRNA in MSC544 and MDA-MB-231 mono-cultures, and in MSC544/MDA-MB-231 co-cultures. Following culture for the indicated times and stimulation for eight days (8 d) with 5 µM SB431542 (SB, only MSC544), the cells were lysed and subjected to RNA isolation and quantitative PCR (qPCR) analyses of TGF-β1. Data were normalized to three housekeeping genes (β-actin, GAPDH, and TBP) amplified from the same sample and are the mean + s.d. from a representative experiment. The asterisks indicate significance relative to the respective 1 d culture (*p* < 0.05, unpaired two-tailed student’s *t*-test).

Both, uPA and PAI-1 are the target genes of TGF-β1 and important mediators of TGF-β bioactivation and proinvasive/prometastatic function [29]. Therefore, it was of interest to determine its presence and possible function in MSC and MDA-MB-231 cultures. The release of bioactive TGF-β1 was quantified in supernatants from MSC544 and MDA-MB-231 mono-cultures, respectively, and in a corresponding co-culture between the MSC544 and MDA-MB-231 cells. While TGF-β1 release from 10,000 MDA-MB-231 cells/24-well remained below detection limit of the ELISA, a similar cell number of 10,000 MSC544/24 well produced 5.9 ± 2.2 pg/mL TGF-β1 (*n* = 3) in the supernatant after 24 h and 22.4 ± 0.5 pg/mL (*n* = 3) TGF-β1 after 72 h (Figure 5). The supernatants that were derived from MSC544 mono-cultures as compared to those that were obtained after co-culture of MSC544 together with MDA-MB-231 cells revealed progressively higher levels of TGF-β1 in the co-cultures (1.56-fold after 24 h and 2.19-fold after 72 h) (Figure 5). We performed qPCR analyses of TGF-β1 in MSC544 and MDA-MB-231 cells that were cultured for one, three, and eight days to reveal whether the increase in secreted protein is due to enhanced mRNA synthesis. To this end, TGF-β1 mRNA levels in MSC544 cells strongly increased with time in culture (15.56-fold from day 1 to day 8). In contrast, the TGF-β1 mRNA abundance was decreased by ~90% in MDA-MB-231 cells on day 3 and day 8 when compared to day 1 (Figure 6). However, little, if any, increase in TGF-β1 mRNA levels was detectable in the co-cultures, which may be due to progressive overgrowing of the MSC544 cells by the (non-TGF-β1 expressing) MDA-MB-231 cells. Moreover, the different turnover kinetics of secreted TGF-β1 protein (which accumulates in the supernatant) and mRNA (which is maintained in a steady-state by rapid synthesis/degradation) contribute to this effect. Finally, the strong increase in TGF-β expression and secretion in MSC544 mono-cultures and TGF-β1 secretion in the co-cultures prompted us to analyze whether auto-regulation may operate in these cultures, since TGF-β1 has been shown to be capable of auto-inducing its own synthesis [30]. As TGF-β1 binding and the activation of the TGF-β type I receptor is a prerequisite for this to occur, we inhibited its kinase activity with the small molecule SB431542 [31] in MSC544 cells mono-cultured for eight d. Intriguingly, the TGF-β1 mRNA levels were suppressed by 59.5% relative to vehicle-treated control (Figure 6).

These findings suggest that (i) MSC, rather than the breast cancer cells, are responsible for TGF-β1 synthesis, (ii) the upregulation of TGF-β1 expression during mono- and co-culture of MCS544 cells may involve a positive autocrine feedback loop, and (iii) the effects on TGF-β1 secretion are synergistic between both populations in the co-cultures.

The potential synergistic effects were also detectable following a time course of uPA and PAI-1 production. Thus, PAI-1 protein levels continuously decreased between day 1 and day 8 in MDA-MB-231 mono-cultures and similar effects were monitored in the co-cultures, while little, if any, changes occurred in MSC544 mono-cultures between day 1 and day 8 (Figure S2, lower panel). Moreover, the amount of uPA protein in MDA-MB-231 and MSC544 mono-cultures remained nearly unchanged, but continuously increased in the co-cultures between day 1 and day 8 (Figure S2, upper panel), which suggested alterations due to mutual interactions between MSC544 and the breast cancer cells until reaching the levels that were observed during long-term co-culture and 3D tumor spheroid formation.

## 3. Conclusions

The present data demonstrate that long-term co-culture of human MSC with the breast cancer cell line MDA-MB-231 alters uPA, PAI-1, and TGF-β1 expression, which are three factors that favor cancer cell dissemination and the increased formation of metastasis. For these studies, MSC544 represent a suitable cellular model because of prominent MSC marker expression, even after long-term culture, exhibition of appropriate MSC-like morphology, and expression of uPA and PAI-1 at similar levels as in primary human MSC cultures. Accordingly, the co-culture results suggest an interactive role for uPA, PAI-1, and TGF-β1 in promoting cancer cell growth.

## 4. Materials and Methods

### 4.1. Cell Culture

Primary human mesenchymal stroma/stem-like cells (MSC) were isolated from umbilical cord tissue explant cultures, as described previously [32]. Briefly, MSC were cultured in MSC growth medium (αMEM (Sigma Chemie GmbH, Steinheim, Germany) that was supplemented with 10% allogeneic human AB-serum, 100 U/mL penicillin, 100 µg/mL streptomycin, and 2 mM l-glutamine (Sigma Chemie GmbH)) and subculture in passages (P) was performed following treatment with accutase (Capricorn Scientific GmbH, Ebsdorfergrund, Germany) at 37 °C for 3 min. The Ethics Committee of Hannover Medical School, Project #443 on February 26th, 2009 approved the use of human MSC and informed written consent was obtained from each patient. For the experiments, the MSC cultures were used from six different donors: MSC081113 P5, MSC030816 P5, MSC100314 P4, MSC290115 P3, MSC280416 P5, and MSC544 P32

Human MDA-MB-231 breast carcinoma cells were commercially obtained from American Type Culture Collection. The triple negative breast cancer cell line MDA-MB-231 was grown in Leibovitz’s L-15-medium (Life Technologies, Darmstadt, Germany), supplemented with 10 % FCS, 100 U/mL penicillin, 100 µg/mL streptomycin, and 2 mM L-glutamine (Sigma Chemie GmbH).

All of the cells were tested for mycoplasma by the luminometric MycoAlert Plus mycoplasma detection kit (Lonza Inc., Rockland, ME, USA), according to the manufacturer’s recommendations. Previous work confirmed the authentication of the breast carcinoma cell line by short tandem repeat (STR) fragment analysis using the GenomeLab human STR primer set (Beckman Coulter Inc., Fullerton, CA, USA).

The stable transduction of MSC544 and MDA-MB-231 cells for discrimination in co-culture was performed with a third generation lentiviral SIN vector carrying either the mCherry gene (MDA-MB-231^cherry^) or the enhanced green fluorescent protein (eGFP) gene (MSC^GFP^). Co-cultures of MSC544^GFP^ and MDA-MB-231^cherry^ were cultivated in MSC growth medium at a cell ratio of 1:1.

### 4.2. Flow Cytometry Analysis

The samples were first blocked with 2% FCS in PBS for 15 min. at room temperature for flow cytometric analysis. Following a PBS washing step, the cells were stained with a PE-labeled monoclonal mouse anti-human CD73 antibody (clone AD2) (BD Bioscience GmbH), a PE-labeled monoclonal CD90 antibody (clone 5E10) (BioLegend via Biozol GmbH, Eching, Germany), or a PE-labeled monoclonal mouse anti-human CD105 antibody (clone 43A3) (BioLegend via Biozol GmbH) at 4 °C for 15 min. A PE-labeled IgG1 antibody for CD73 or a double-labeled PE/FITC IgG1 antibody (both from Dako Denmark AS, Glostrup, Denmark) served as an appropriate control. Thereafter, the cells were washed again with PBS and subsequently analyzed by flow cytometry using FACScalibur (BD Biosciences GmbH, Heidelberg, Germany) and then analyzed by FlowJo V10 software.

### 4.3. Quantification of Human Urokinase Plasminogen Activator (uPA) and Human Plasminogen Activator Inhibitor-1 (PAI-1) by ELISA

A quantitative determination of uPA and PAI-1 in the protein homogenates of MDA-MB-231 breast cancer cells, the different MSC cell populations, and a homogenate of 3D spheroids from a MSC544^GFP^/MDA-MB-231^cherry^ co-culture was performed by appropriate enzyme-linked immunosorbent assays (ELISA), as described previously [27]. Briefly, the cell cultures and the 3D spheroid co-culture were frozen in liquid nitrogen and pulverized in the frozen state while using a Sartorius microdismembrator S (Fisher Scientific GmbH, Schwerte, Germany). The resulting powder was suspended in extraction buffer (American Diagnostica GmbH, Pfungstadt, Germany) and the determination of protein content was performed by the colorimetric BCA-assay (Perbio Science Deutschland, Bonn, Germany). Appropriate protein aliquots were applied to the uPA and PAI-1 ELISA, respectively, by following a protocol according to the manufacturer’s instructions (American Diagnostica GmbH). The detection limit is 25 pg/mL for the uPA ELISA and 125 pg/mL for the PAI-1 ELISA.

### 4.4. TGF-β1 ELISA

MSC544 P17 were plated in 24-well-plates at 10,000 cells/well in 1 mL/well of MSC growth medium. A co-culture of 5,000 MSC544 P17, together with 5,000 MDA-MB-231 cells in 24-well with 1 mL of MSC growth medium, was cultured in parallel. Following cell attachment, the cultures were washed twice with serum-free αMEM, and thereafter incubated in αMEM supplemented with 0.1% allogeneic human AB-serum for 24 and 72 h. At these times, ELISA removed the aliquots of the cell culture supernatants for the quantification of bioactive TGF-β1. Plain αMEM that was supplemented with 0.1% allogeneic human AB-serum and incubated for 24 and 72 h in a 37 °C humidified atmosphere served as medium control. Following appropriate dilution, the supernatants were subjected to TGF-β1-specific ELISA (Human/Mouse TGF β1 ELISA Ready-SET-Go!, eBioscience/Affymetrix Inc. San Diego, CA, USA), according to the manufacturer’s instructions and Ref. [33]. The detection limit of this ELISA is 25 pg/mL.

### 4.5. qPCR Analysis

Total RNA was purified from MDA-MB-231 and MSC544 cells, and the co-cultures using the RNeasy Mini Kit (Qiagen, Hilden, Germany), according to manufacturer’s recommendations. One µg RNA of each sample was subjected to reverse transcription for 1 h at 37°C with M-MLV Reverse Transcriptase (200 U, Life Technologies) and random hexamers (2.5 µM, Life Technologies) in a total volume of 20 µL. The relative mRNA concentrations of the target genes were quantified by qPCR on an I-Cycler (BioRad, Hercules, CA, USA) with Maxima SYBR Green Mastermix (Thermo Fisher Scientific, Waltham, MA, USA). For each sample, the Ct values for the target genes were normalized to those for TATA box-binding protein (TBP). The following sense (s) and antisense (as) PCR primers were used (5’–3’): EGF-s: tggatgtgcttgataagcgg, EGF-as: accatgtcctttccagtgtgt; CCL5/RANTES-s: ccagcagtcgtctttgtcac, CCL5/RANTES-as: ctctgggttggcacacactt; TGF-β1-s: cccagcatctgcaaagctc, TGF-β1-as: gtcaatgtacagctgccgca; β-actin-s: gacgaggcccagagcaagag, β-actin-as: atctccttctgcatcctgtc; GAPDH-s: ttgccatcaatgaccccttca, GAPDH-as: cgccccacttgattttgga; and, TBP-s: gctggcccatagtgatcttt, TBP-as: cttcacacgccaagaaacag.

## Supplementary Materials

The following are available online at https://www.mdpi.com/1422-0067/20/11/2630/s1.

## Abbreviations

eGFP EGF CCL5	enhanced green fluorescent protein epidermal growth factor C-C motif chemokine ligand 5
FACS	fluorescence-activated cell sorting
FCS	fetal calf serum
MSC	mesenchymal stroma/stem-like cells
P	passage
PAI-1	plasminogen activator inhibitor-1
TGF-β uPA	transforming growth factor-β urokinase plasminogen activator

## References

[1] Brabek J., Mierke C.T., Rosel D., Vesely P., Fabry B. (2010). The role of the tissue microenvironment in the regulation of cancer cell motility and invasion. Cell Commun. Signal.

[2] Leung C.T., Brugge J.S. (2009). Tumor self-seeding: Bidirectional flow of tumor cells. Cell.

[3] Dittmer J. (2010). Mesenchymal stem cells: “repair cells” that serve wounds and cancer?. Sci. World J..

[4] Hass R., Otte A. (2012). Mesenchymal stem cells as all-round supporters in a normal and neoplastic microenvironment. Cell Commun. Signal.

[5] Tang L., Han X. (2013). The urokinase plasminogen activator system in breast cancer invasion and metastasis. Biomed. Pharmacother..

[6] Carter J.C., Church F.C. (2009). Obesity and breast cancer: The roles of peroxisome proliferator-activated receptor-gamma and plasminogen activator inhibitor-1. PPAR Res..

[7] Lampelj M., Arko D., Cas-Sikosek N., Kavalar R., Ravnik M., Jezersek-Novakovic B., Dobnik S., Dovnik N.F., Takac I. (2015). Urokinase plasminogen activator (uPA) and plasminogen activator inhibitor type-1 (PAI-1) in breast cancer - correlation with traditional prognostic factors. Radiol. Oncol..

[8] Ikushima H., Miyazono K. (2010). TGFbeta signalling: A complex web in cancer progression. Nat. Rev. Cancer.

[9] Yu Q., Stamenkovic I. (2000). Cell surface-localized matrix metalloproteinase-9 proteolytically activates TGF-beta and promotes tumor invasion and angiogenesis. Genes Dev..

[10] Yang Y., Otte A., Hass R. (2015). Human mesenchymal stroma/stem cells exchange membrane proteins and alter functionality during interaction with different tumor cell lines. Stem Cells Dev..

[11] Kulterer B., Friedl G., Jandrositz A., Sanchez-Cabo F., Prokesch A., Paar C., Scheideler M., Windhager R., Preisegger K.H., Trajanoski Z. (2007). Gene expression profiling of human mesenchymal stem cells derived from bone marrow during expansion and osteoblast differentiation. BMC Genom..

[12] Otte A., Bucan V., Reimers K., Hass R. (2013). Mesenchymal stem cells maintain long-term in vitro stemness during explant culture. Tissue Eng. Part C Methods.

[13] Yang Y., Melzer C., Bucan V., von der Ohe J., Otte A., Hass R. (2016). Conditioned umbilical cord tissue provides a natural three-dimensional storage compartment as in vitro stem cell niche for human mesenchymal stroma/stem cells. Stem Cell Res. Ther..

[14] Melzer C., von der Ohe J., Hass R. (2019). In vivo cell fusion between mesenchymal stroma/stem-like cells and breast cancer cells. Cancers.

[15] Dittmer A., Hohlfeld K., Lutzkendorf J., Muller L.P., Dittmer J. (2009). Human mesenchymal stem cells induce E-cadherin degradation in breast carcinoma spheroids by activating ADAM10. Cell. Mol. Life Sci..

[16] Klopp A.H., Lacerda L., Gupta A., Debeb B.G., Solley T., Li L., Spaeth E., Xu W., Zhang X., Lewis M.T. (2010). Mesenchymal stem cells promote mammosphere formation and decrease E-cadherin in normal and malignant breast cells. PLoS ONE.

[17] Jankun J., Skrzypczak-Jankun E. (1999). Molecular basis of specific inhibition of urokinase plasminogen activator by amiloride. Cancer Biochem. Biophys..

[18] Gkretsi V., Stylianou A., Stylianopoulos T. (2017). Vasodilator-Stimulated Phosphoprotein (VASP) depletion from breast cancer MDA-MB-231 cells inhibits tumor spheroid invasion through downregulation of Migfilin, beta-catenin and urokinase-plasminogen activator (uPA). Exp. Cell. Res..

[19] Meng D., Lei M., Han Y., Zhao D., Zhang X., Yang Y., Liu R. (2018). MicroRNA-645 targets urokinase plasminogen activator and decreases the invasive growth of MDA-MB-231 triple-negative breast cancer cells. OncoTargets Ther..

[20] Melzer C., Yang Y., Hass R. (2016). Interaction of MSC with tumor cells. Cell. Commun. Signal..

[21] Melzer C., von der Ohe J., Hass R. (2018). In Vitro Fusion of Normal and Neoplastic Breast Epithelial Cells with Human Mesenchymal Stroma/Stem Cells Partially Involves Tumor Necrosis Factor Receptor Signaling. Stem Cells.

[22] Berndt B., Zanker K.S., Dittmar T. (2013). Cell fusion is a potent inducer of aneuploidy and drug resistance in tumor cell/ normal cell hybrids. Crit. Rev. Oncog..

[23] Li H.J., Reinhardt F., Herschman H.R., Weinberg R.A. (2012). Cancer-stimulated mesenchymal stem cells create a carcinoma stem cell niche via prostaglandin E2 signaling. Cancer Discov..

[24] Melzer C., von der Ohe J., Lehnert H., Ungefroren H., Hass R. (2017). Cancer stem cell niche models and contribution by mesenchymal stroma/stem cells. Mol. Cancer.

[25] Melzer C., Hass R., Lehnert H., Ungefroren H. (2019). RAC1B: A Rho GTPase with Versatile Functions in Malignant Transformation and Tumor Progression. Cells.

[26] Karnoub A.E., Dash A.B., Vo A.P., Sullivan A., Brooks M.W., Bell G.W., Richardson A.L., Polyak K., Tubo R., Weinberg R.A. (2007). Mesenchymal stem cells within tumour stroma promote breast cancer metastasis. Nature.

[27] Mandel K., Seidl D., Rades D., Lehnert H., Gieseler F., Hass R., Ungefroren H. (2013). Characterization of spontaneous and TGF-beta-induced cell motility of primary human normal and neoplastic mammary cells in vitro using novel real-time technology. PLoS ONE.

[28] Ungefroren H., Sebens S., Seidl D., Lehnert H., Hass R. (2011). Interaction of tumor cells with the microenvironment. Cell. Commun. Signal..

[29] Santibanez J.F., Obradovic H., Kukolj T., Krstic J. (2018). Transforming growth factor-beta, matrix metalloproteinases, and urokinase-type plasminogen activator interaction in the cancer epithelial to mesenchymal transition. Dev. Dyn..

[30] Zhang Q., Yu N., Lee C. (2014). Vicious cycle of TGF-beta signaling in tumor progression and metastasis. Am. J. Clin. Exp. Urol..

[31] Inman G.J., Nicolas F.J., Callahan J.F., Harling J.D., Gaster L.M., Reith A.D., Laping N.J., Hill C.S. (2002). SB-431542 is a potent and specific inhibitor of transforming growth factor-beta superfamily type I activin receptor-like kinase (ALK) receptors ALK4, ALK5, and ALK7. Mol. Pharmacol..

[32] Mandel K., Yang Y., Schambach A., Glage S., Otte A., Hass R. (2013). Mesenchymal stem cells directly interact with breast cancer cells and promote tumor cell growth in vitro and in vivo. Stem Cells Dev..

[33] Witte D., Otterbein H., Forster M., Giehl K., Zeiser R., Lehnert H., Ungefroren H. (2017). Negative regulation of TGF-beta1-induced MKK6-p38 and MEK-ERK signalling and epithelial-mesenchymal transition by Rac1b. Sci. Rep..

