# Supplementary material for: Changes in uPA, PAI-1, and TGF-β Production during Breast Cancer Cell Interaction with Human Mesenchymal Stroma/Stem-Like Cells (MSC)
Source: Int J Mol Sci. 2019 May 28;20(11):2630. doi: 10.3390/ijms20112630 (PMC6600334; doi:10.3390/ijms20112630)
Supplement: Supplementary file 1 [file ijms-20-02630-s001.pdf]

International Journal of
Molecular Sciences

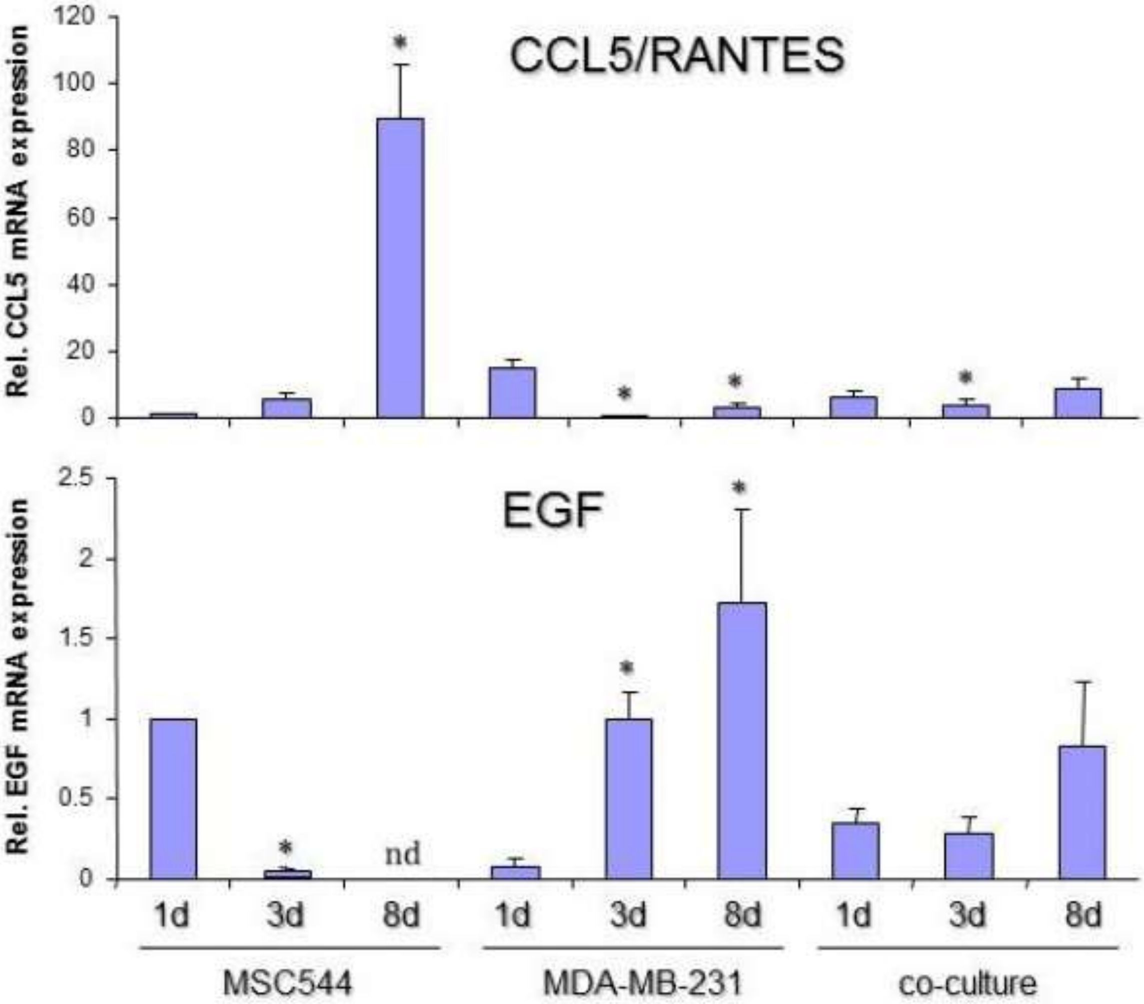

Figure S1. Quantification of CCL5/RANTES (upper panel) and EGF-specific mRNA (lower panel) in MSC544 and MDA-MB-231 mono-cultures, and in MSC544/MDA-MB-231 co-cultures. Following culture for the indicated times, cells were lysed and subjected to RNA isolation and qPCR analyses of CCL5 and EGF, respectively. Data were normalized to three housekeeping genes (beta-actin, GAPDH, and TBP) amplified from the same sample and represent the mean + s.d. from a representative experiment. The asterisks indicate significance relative to the respective 1d culture ($p < 0.05$, unpaired two-tailed student's t test).

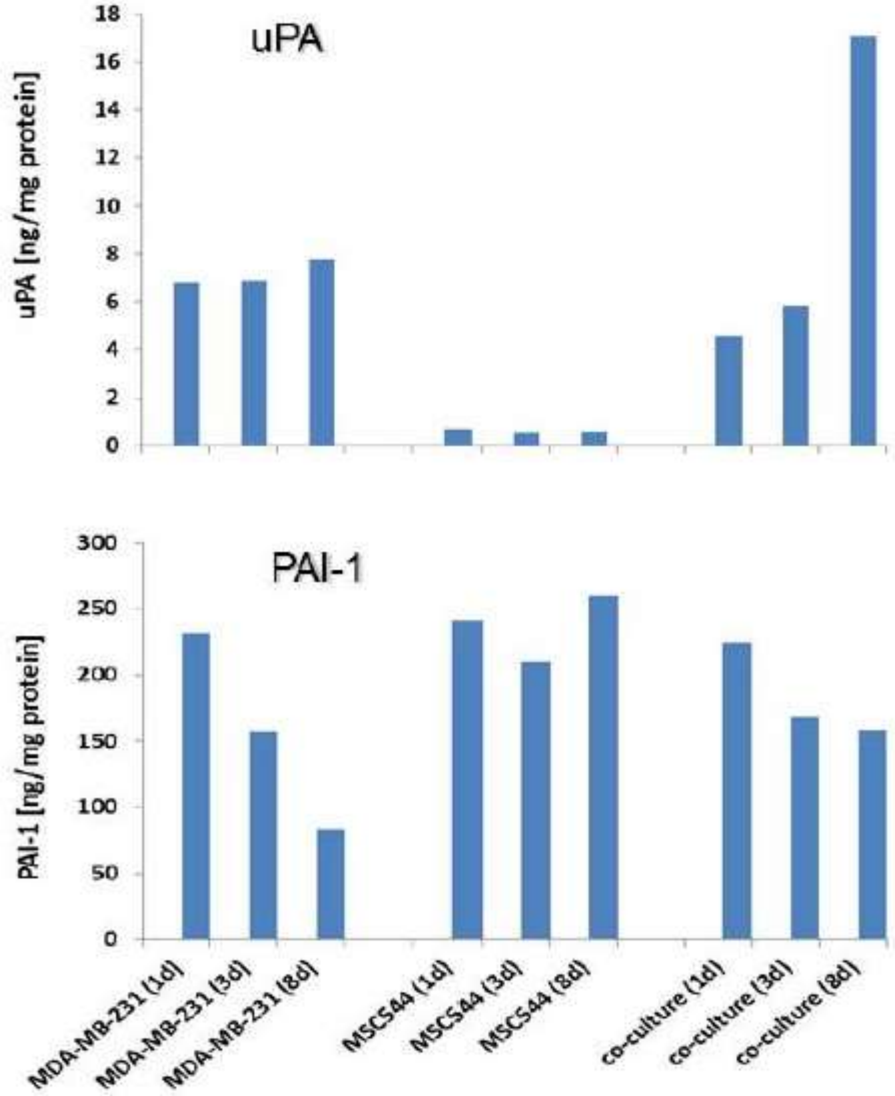

Figure S2. Time course of intracellular uPA (upper panel) and PAI-1 (lower panel) protein production by MDAMB-231^{cherry} breast cancer cells, MSC544^{GFP} P34, and MSC544/MDA-MB-231 co-cultures for 1 day, 3 days, and 8 days, respectively. Intracellular protein levels of uPA and PAI-1 were evaluated as the mean of duplicate measurements following homogenization of the cell populations and protein quantification.

© 2019 by the authors. Licensee MDPI, Basel, Switzerland. This article is an open access article distributed under the terms and conditions of the Creative Commons Attribution (CC BY) license (http://creativecommons.org/licenses/by/4.0/).
